# Plant Stress Granules: Trends and Beyond

**DOI:** 10.3389/fpls.2021.722643

**Published:** 2021-08-09

**Authors:** Israel Maruri-López, Nicolás E. Figueroa, Itzell E. Hernández-Sánchez, Monika Chodasiewicz

**Affiliations:** Biological and Environmental Science and Engineering Division, Center for Desert Agriculture, King Abdullah University of Science and Technology, Thuwal, Saudi Arabia

**Keywords:** plant stress granules, phase separation, intrinsically disordered regions, RNA-binding domains, small molecules, preexisting complex, post-translational modifications, four-phase assembly model

## Abstract

Stress granules (SGs) are dynamic membrane-less condensates transiently assembled through liquid–liquid phase separation (LLPS) in response to stress. SGs display a biphasic architecture constituted of core and shell phases. The core is a conserved SG fraction fundamental for its assembly and consists primarily of proteins with intrinsically disordered regions and RNA-binding domains, along with translational-related proteins. The shell fraction contains specific SG components that differ among species, cell type, and developmental stage and might include metabolic enzymes, receptors, transcription factors, untranslated mRNAs, and small molecules. SGs assembly positively correlates with stalled translation associated with stress responses playing a pivotal role during the adaptive cellular response, post-stress recovery, signaling, and metabolic rewire. After stress, SG disassembly releases mRNA and proteins to the cytoplasm to reactivate translation and reassume cell growth and development. However, under severe stress conditions or aberrant cellular behavior, SG dynamics are severely disturbed, affecting cellular homeostasis and leading to cell death in the most critical cases. The majority of research on SGs has focused on yeast and mammals as model organism. Nevertheless, the study of plant SGs has attracted attention in the last few years. Genetics studies and adapted techniques from other non-plant models, such as affinity capture coupled with multi-omics analyses, have enriched our understanding of SG composition in plants. Despite these efforts, the investigation of plant SGs is still an emerging field in plant biology research. In this review, we compile and discuss the accumulated progress of plant SGs regarding their composition, organization, dynamics, regulation, and their relation to other cytoplasmic foci. Lastly, we will explore the possible connections among the most exciting findings of SGs from mammalian, yeast, and plants, which might help provide a complete view of the biology of plant SGs in the future.

## A Brief History of Stress Granules

Cells are highly dynamic systems that are continuously subjected to fluctuating environments (Kollist et al., 2019). To respond, adapt, and ultimately survive, cells rapidly rewire their transcriptome, metabolome, proteome, and degradome profiles (Kollist et al., 2019). Remarkably, messenger RNA (mRNA) metabolism is crucial for growth, development, and stress responses. These processes require the assembly of mRNA-ribonucleoprotein (mRNP) complexes, such as polysomes, processing bodies (PBs), and stress granules (SGs) (Chantarachot and Bailey-Serres, 2018).

Nover et al. (1983) described the presence of granular cytoplasmic aggregates in heat-treated tomato cell cultures. These aggregates were mainly composed of heat-shock proteins (HSPs) and untranslated mRNAs (Nover et al., 1989), and were called as heat-shock granules (HSGs). Afterward, Collier and Schlesinger (1986) and Arrigo et al. (1988) observed the presence of granules composed of HSPs in chicken embryo fibroblasts and HeLA cells exposed to heat stress, respectively. Then, Kedersha et al. (1999) reported the conditions triggering the formation of mammalian SGs and their primary composition (polyA + mRNA and core proteins) and defined them as the cytoplasmic foci at which untranslated mRNAs accumulate in response to stress, placing them as the counterparts of the plant SGs as described by Nover et al. (1983). Thus, similar foci were reported in yeast cells under glucose starvation, heat, and oxidative stress (Hoyle et al., 2007; Buchan et al., 2008; Groušl et al., 2009).

Subsequently Weber et al. (2008), using immunofluorescence analyses, described the presence of three unambiguously different stress-related cytoplasmic granules in plant cells to classify them according to different compositions and to assemble kinetics into: (1) HSGs, (2) SGs, and (3) PBs. SGs occur after short-term heat treatment, whereas HSGs are formed under long-term heat stress condition. Moreover, HSGs do not contain polyA + mRNA as the association of mRNAs with HSGs mentioned by Nover et al. (1989) corresponds to an artifact of co-sedimentation of SGs and HSGs in the isolation procedure (Weber et al., 2008). The features of plant SGs, as a rapid assembling under heat stress and containing polyA + mRNA, situate plant SGs as a real equivalent of SGs reported in human cells by Kedersha et al. (1999). The third foci, called as PBs, are constitutive mRNP granules associated with translation repression and mRNA decay as they contain mRNA degradation factors, namely DCP1, DCP2, and XRN4 (Weber et al., 2008; Chantarachot and Bailey-Serres, 2018).

A lot of research on SGs has been carried out in mammalian models in which the alterations in their assembly or disassembly are linked to several degenerative diseases (Ramaswami et al., 2013; Wolozin and Ivanov, 2019). Recent data have shown that plant SGs, like in mammalian cells, might also be involved in response to viral infection, which denote the importance of studying the cellular and physiological role of plant SG biology (Lloyd, 2012; Krapp et al., 2017).

## Composition of SGs: Recruited Proteins, RNA, and Small Molecules

Despite their significance for plant cell biology, the composition of plant SGs has been poorly described. In the last few years, studies focusing on the use of SG markers as a bait coupled to high-throughput chromatography approaches have significantly powered the knowledge about the plant SG composition, revealing conserved, novel, and plant-specific RNAs, proteins, and metabolites (Takahara and Maeda, 2012; Sorenson and Bailey-Serres, 2014; Bhasin and Hülskamp, 2017; Kosmacz et al., 2019; Gutierrez-Beltran et al., 2020).

The composition of SGs is heterogeneous, and the presence and distribution of their different elements can change considerably due to different types of stresses (Jain et al., 2016; Wheeler et al., 2016; Markmiller et al., 2018; Niewidok et al., 2018; Kosmacz et al., 2019; Guillén-Boixet et al., 2020). In mammalian and yeast cells, extensive studies using fluorescence *in situ* hybridization (FISH), affinity purification (AP), proximity labeling (PL), and mass spectrometry, etc., had helped to investigate the composition and dynamics of SGs (Jain et al., 2016; Markmiller et al., 2018; Youn et al., 2018). In the following sections, we examine the most recent progress regarding the proteins, RNAs, and metabolite composition in plant SGs.

## SG-Associated Proteins

To date, approximately 500 proteins have been annotated in the mammalian SG proteome database (Nunes et al., 2019). The SG proteome is mainly composed of intrinsically disordered proteins (IDPs) or proteins containing intrinsically disordered regions (IDRs), RNA−binding proteins, prion-like domains (PrLD) containing proteins, and proteins with ATPase activity. SG proteins are categorized in detail into translation initiation complex-related proteins, proteins associated with RNA processing, and spliceosome subunit proteins, which are enriched in disordered protein regions, along with ATP-dependent remodeling complexes (Kedersha and Anderson, 2002; Kedersha et al., 2005; Banani et al., 2017; Markmiller et al., 2018; Youn et al., 2018, 2019). Integral components of the SG cores in yeast and mammalian cells are the 40S ribosomal subunit, eukaryotic translation initiation factors eIF3 and 4G, poly(A)-binding protein cytoplasmic 1 (PAB1), Ras-GAP SH3 domain-binding protein (G3BP1 and G3BP2), and prion-related RNA-binding protein (TIA-1), ubiquitin-associated protein 2-like (UBAP2L), etc. (Gilks et al., 2004; Jain et al., 2016; Kedersha et al., 2016; Huang et al., 2020).

About one-fourth of the identified SG-localized proteins in plants have a known ortholog in either human or yeast SGs (Jain et al., 2016; Markmiller et al., 2018; Youn et al., 2018; Kosmacz et al., 2019). Therefore, the described plant SG proteins have only been identified based on their homology with animal and yeast proteins. For instance, the *Arabidopsis thaliana* SG markers, the RNA-binding protein 47b (Rbp47b) and oligouridylate binding protein 1B (UBP1B), are the RNA-binding proteins most closely related to mammalian TIA-1, which is an initial component during human SG assembly (Kedersha et al., 1999; Gilks et al., 2004). Rbp47b and UBP1B are considered as the core elements of plant SGs. Interestingly, under normal conditions, these proteins exhibit cytoplasmic and/or nuclear localization, whereas stress treatment induces shuttling into the SG foci (Table 1). The conserved function of these proteins in SG assembly suggests an involvement in an evolutionarily conserved mechanism to face stress (Jain et al., 2016; Chantarachot and Bailey-Serres, 2018).

**TABLE 1:** Stress granule (SG) formation and stress tolerance.

**Protein name**	**ID**	**GO Molecular Function**	**Normal subcellular localization**	**Nature of stress**	**References**
Rbp47b	At1g19130	mRNA binding, poly(A) binding	Cytoplasm and nucleus	Heat	Weber et al., 2008
Ubp1B	At1g17370	mRNA binding	Nucleus	Heat, salt, and osmotic	Weber et al., 2008; Nguyen et al., 2016, 2017
Ubp1C	At3g14100	mRNA binding	Cytoplasm and nucleus	Hypoxia	Sorenson and Bailey-Serres, 2014
TSN1	At5G07350	mRNA catabolism	Cytoplasm	Heat and salt	Yan et al., 2014; Gutierrez-Beltran et al., 2015
TSN2	At5G61780	mRNA catabolism	Cytoplasm	Heat and salt	Yan et al., 2014; Gutierrez-Beltran et al., 2015
ANGUSTIFOLIA	At1G01510	Transcriptional regulation and membrane trafficking	Cytosol and trans-Golgi network	Heat, salt, osmotic, and hypoxia	Bhasin and Hülskamp, 2017
G3BP-2	At5G43960	mRNA binding	Cytoplasm and nucleus	Heat and pathogens	Krapp et al., 2017; Reuper et al., 2021
HSP101	At1G74310	Chaperone, ATPase	Cytoplasm	Heat	Merret et al., 2017; McLoughlin et al., 2019
RH6, RH8, and RH12	AT2G45810 AT4G00660 AT3G61240	Helicase	Cytoplasm and nucleus	Hypoxia	Chantarachot et al., 2020

Pioneering studies have explored the protein composition of *A. thaliana* SGs using the angustifolia (AN) protein as a bait. AN is recruited in SGs under heat stress, salt stress, osmotic stress, and low-oxygen stress (Bhasin and Hülskamp, 2017). The protein interactome screening was analyzed by mass spectrometry and revealed that AN is associated with numerous SG protein components, including Rbp47 and 45, eukaryotic initiation factor (eIF4E1), tandem zinc finger 3 (TZF3), poly(A)binding protein 2 (PAB2), etc. Interestingly, AN interactions with some SG components are also seen under non-stress conditions (Bhasin and Hülskamp, 2017).

Furthermore, the protein composition of *A. thaliana* SGs formed under heat/dark conditions has been explored through affinity purification mass spectrometry (AP-MS) approaches against GFP-tagged Rbp47b expressing lines (Kosmacz et al., 2019). Analysis of prey identified 118 proteins where a fraction of proteins corresponded to the conserved SG components such as RNA processing and disordered region-containing proteins. Another fraction of proteins unveiled enzymes with posttranslational modification (PTM) activities, such as cyclin-dependent kinase A;1 (CDKA;1), mitogen-activated MKK5 and MPK3 kinases, SNF1-related protein kinase (SnRK) 2.1, as well as reactive oxygen species-related enzymes, including glutathione-*S*-transferases, glutathione peroxidase, and ascorbate peroxidases. Furthermore, elements related to sugar metabolism (Rhamnose RHM1, RHM2, and UER1 enzymes), and ethylene biosynthetic enzymes (ACC oxidases 2 and 4), were well represented. These constitute new SG-related proteins, which are not previously described in plants (Kosmacz et al., 2019).

Tudor staphylococcal nuclease (TSN) proteins have been used to resolve the proteome of *A. thaliana* SGs under heat stress. TSN was identified as a core component of plant SGs (Yan et al., 2014; Gutierrez-Beltran et al., 2015). TSN2 interactome analysis yielded 315 and 176 proteins under no treatment and stress conditions, respectively. The well-known SG proteins in mammals, yeast, and plant are the enriched most TSN-interacting proteins. Remarkably, novel constituents, such as RNA-binding proteins with IDRs and proteins with ATPase activities (plant-specific PAB4, Rbp47b, and SnRK1), were found. Some of these protein interactions were found in a stress-dependent fraction. For instance, Rbp47b, UBP1C, and PAB4 are the components of TSN SGs under non-stress conditions. The HSP70 and SnRK1 kinase have been described as stress-dependent TSN2-interactors (Gutierrez-Beltran et al., 2020). Lectin ArathEULS3 interactome analysis in *A. thaliana* revealed the presence of translational elongation factors, ribosomal, RNA-binding, and HSPs (Dubiel et al., 2020).

Recently, in parallel to cytoplasmic SG assembly, the formation of independent nuclear SG-like structures in tellurite-treated U2OS human cells in a time- and dose-dependent manner has been reported (Gaete-Argel et al., 2021). Interestingly, these nuclear SG-like foci contain the well-known mammalian cytoplasmic SG components G3BP-1 and eIF3b and are different from the previously reported nuclear speckles [reviewed in Lamond and Spector (2003) and Galganski et al. (2017)] since the number and localization of nuclear speckles markers were not altered by tellurite treatment. If plants are also able to form these nuclear SGs under certain stress conditions, is something that remains to be elucidated.

**FIGURE 1:**
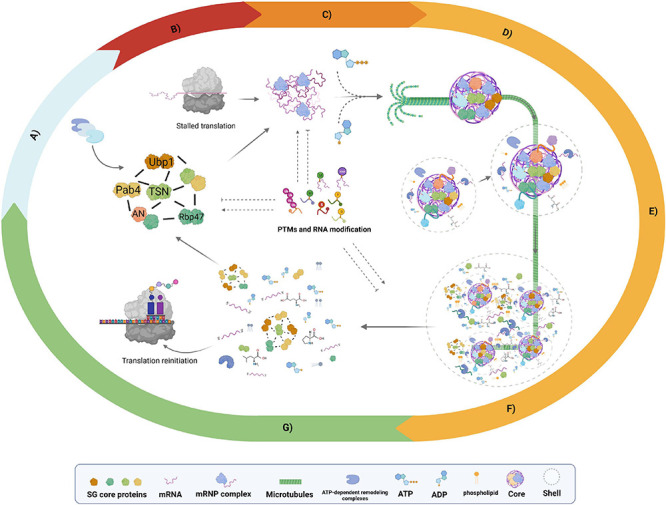
Proposed model stress granule (SG) assembly and disassembly. Schematic representation of SG dynamics based on accumulated evidence of non-plant and plant models. **(A)** Preexisting protein network, experimental evidence revealed protein–protein interaction (PPI) networks of SG core proteins such as RNA-binding protein 47b (Rbp47), tudor staphylococcal nuclease (TSN), PAB4, and angustifolia (AN) proteins under non-stress conditions. **(B)** Stalled translation, a global reduction in translation under stress response generates an mRNA-ribonucleoprotein (mRNP) influx essential for SG assembly. **(C)** Nucleation, high concentrations of mRNP induce liquid–liquid phase separation (LLPS) of mRNP complexes. **(D)** Core growth, the recruitment of additional SG components to nucleated mRNP drives the establishment of visible core structures; this phase is an ATP- and microtubule (MT)-dependent process. **(E)** Shell growth phase, once the core structure is defined, additional mRNPs, specific proteins, small molecules, nucleotides, amino acids, and phospholipids are recruited as shell components. **(F)** Fusion phase, after the formation of individual SGs, fusion events with adjacent SGs take place to assemble a multicore structure immersed in a single shell. **(G)** SG disassembly occurs after stress during the recovery period. The SGs begin with shell dissociation, followed by the core disassembly, the ATP-dependent remodeling complexes are crucial in this step. The upper box denotes the elements presents in the scheme. Posttranslational modifications (PTMs) and RNA modifications: Ub, ubiquitination; M, methylation; P, phosphorylation; S, SUMOylation; Par, PARylation. Dashed lines indicate confirmed evidence in non-plant models that could also occur in plants but is not yet explored. Created with BioRender.com.

Stress granule-like foci (chSGs) assemblies were intriguingly reported inside the chloroplasts of a unicellular green algae *Chlamydomonas reinhardtii* during oxidative stress. The chSGs contain the similar components of cytosolic SG, such as disassembled polysomes, poly(A)-binding, and small ribosomal subunit proteins (Uniacke and Zerges, 2008). Similarly, heat-induced SG-like structures (cpSGs) were observed in *A. thaliana* chloroplasts. The snowy cotyledon 1 protein (SCO1), a plastidial translation elongation factor, was identified as a plastidial protein marker for cpSGs in response to heat stress (Chodasiewicz et al., 2020). An analysis of SCO1-cpSGs proteome interactions revealed RNA-binding proteins containing IDRs, chaperones, and translational elongation factors. Notably, CP29A and DEAD-box RNA helicase (RH3), HSP90-5, and translation elongation factor Tu (RABE1b) were found to be the key components of cpSGs. Moreover, Rubisco activase and ribulosebisphosphate carboxylase/oxygenase (Rubisco) accumulation factors were also present in heat-induced cpSGs from *A. thaliana* (Chodasiewicz et al., 2020). These foci represent a novel class of SGs described in plant plastid organelles. So far, those reports have significantly contributed to extending the catalog of proteins associated to plant SGs. Nevertheless, many biological aspects of SGs remain undefined, particularly those regarding their assembly, stress-, and tissue-specific composition, and structural organization (Youn et al., 2018). The existence of some of those interactomes under non-stress conditions brings to the light the presence of a preexisting protein network among core components (Figure 1A).

## SG-Associated mRNAs

Besides proteins, mammalian SGs preferentially contain some long-non-coding RNAs (lncRNA) and translationally stalled mRNAs with long coding and untranslated regions (UTRs) (Khong et al., 2017). Purification, FISH, and RNA-seq techniques have been applied to investigate the dynamics of mRNAs in SGs. The results showed that mRNAs are shuttled into mammalian SGs in a non-specific way (Stöhr et al., 2006). In the functional level, most of the SG-sequestered transcripts encode housekeeping ribosomal genes. By contrast, mRNAs of stress response-related genes, such as *HSP70* and multidrug resistance 1 (*MDR1*), fail to accumulate in SGs; other genes, such as *cyclin kinase inhibitor p21*, are concentrated into mammalian SGs in a stress-dependent way (Lian and Gallouzi, 2009; Unworth et al., 2010; Yagüe and Raguz, 2010; Silver and Noble, 2012). While many SG-associated mRNAs have been identified in mammalian cells, the association mechanism of RNA and SG in plants remains elusive (Namkoong et al., 2018; Wilbertz et al., 2019; Tian et al., 2020).

Previous studies have recognized the functional outcome of mRNAs and SG-associated proteins. In the case of *A. thaliana* UBP1B-SGs, the transcripts encoding for the *DnaJ* (a HSP) and the stress-associated protein (*AtSAP3*) were reported as the targets of UBP1B protein, which are preferentially stored in SGs under heat stress (Nguyen et al., 2016). Under low-oxygen stress, UBP1C-SGs sequestrate mRNAs with uracil-rich 3′-UTRs by a direct interaction. Thus, the hypoxia-responsive mRNAs are preferentially translated. After hypoxia, SGs disassemble, and captive mRNAs return to polysomes (Sorenson and Bailey-Serres, 2014).

Similarly, *A. thaliana* PABP2-SGs preferentially protect the ribosomal proteins encoded by mRNAs from heat-shock stress. During recovery, these ribosomal mRNAs are released and translated through a mechanism that requires HSP101 (Merret et al., 2017). In the same way, TSN1-SGs protect a specific set of transcripts from degradation under salinity stress, including the mRNA encoding gibberellin (GA) 20-oxidase 3, a key enzyme in GA biosynthesis (Yan et al., 2014).

Messenger RNA recruitment has been observed inside plastidial SGs. Particularly, in *A. thaliana*, the most abundant plastid transcripts were those encoding ribosomal proteins and the subunits of the ATP synthase complex (Chodasiewicz et al., 2020). In *C. reinhardtii*, the recruited mRNAs encode the subunits of photosystem II (PS II; *psbA* and *psbC*), photosystem I (*psaA*), and Rubisco (Uniacke and Zerges, 2008).

## SG-Associated Metabolites

Cell endogenous metabolites are considered as an emergent element of SG biology (Kosmacz et al., 2018, 2019; Begovich et al., 2020; Chodasiewicz et al., 2020). Metabolites that are localized in SGs as chemical molecules may facilitate SG assembly, which might be depending on the accumulation of a specific set of SG-associated proteins. For example, in yeast and mammalian cells, SG proteome revealed several ATP-dependent, lipid-binding proteins, and proteins with amino acid- and nucleotide-binding functions (Kedersha et al., 1999; Jain et al., 2016; Markmiller et al., 2018). Essentially, yeast and mammalian SGs require ATP for their assembly and liquid-like behavior (Jain et al., 2016; Eum et al., 2020). *S*-adenosylmethionine was identified as a regulator of yeast SG assembly and composition (Begovich et al., 2020). Zn^2+^ has been identified as a stress-inducible second messenger that guides mammalian SG assembly and dynamics (Rayman et al., 2018).

The metabolomic analysis of heat-induced plant SGs revealed the presence of nucleotides (adenine dinucleotide phosphate), amino acids (proline, glutamic acid, leucine, and methionine), and phospholipid precursors (citicoline and phosphoethanolamine) (Kosmacz et al., 2019). Subsequently, Chodasiewicz et al. (2020) identified fatty acids, stearic acid, palmitic acid, and the two amino acids, glutamic acid and proline, as the components of cpSGs from *A. thaliana*.

It has been proposed that metabolites are recruited into SGs through small molecule–protein interactions, with small molecules increasing protein thermostability and keeping their folding state (Radauer et al., 2008; Jain et al., 2016; Kosmacz et al., 2018, 2019; Markmiller et al., 2018; Eum et al., 2020). For example, proline accumulated in SGs may activate molecular chaperones and avoid misfolding of proteins localized in SGs (Diamant et al., 2001; Mateus et al., 2016).

## Mechanism of SG Assembly and Disassembly: An Overview of the Research Gaps From the Plant Perspective

Liquid–liquid phase separation (LLPS) is a reversible and highly controlled phenomenon by which proteins and nucleic acids coacervate from the aqueous environment (diluted phase), driving the formation of membrane-less organelle (MLO) structures of micron-scale, which can concentrate 10- to 300-fold molecules more than the surrounding environment (dense phase). SGs exhibit a type of MLO (Banani et al., 2017). Under the condition of stress response, eukaryotic cells experience almost a complete shutdown of translation, leading to polysome disassembly, the early release of mRNA, and the establishment of mRNP complexes (Figure 1B; Hofmann et al., 2020). Biophysical evidence from mammals, yeast, and plants suggests SG assembly and dynamics as a conserved mechanism in eukaryotes driven by LLPS of mRNA complexes in a multistep and tightly controlled process that can be summarized as follows: (1) nucleation, (2) core growth, and (3) shell assembly (growth and fusion phases) (Figures 1C–F; Banani et al., 2017; Markmiller et al., 2018; Youn et al., 2018; Kosmacz et al., 2019; Cirillo et al., 2020). Single-molecule time-course analyses showed that SG assembly requires a nucleation process as a primary step; subsequently, this structure will be directed to a core formation followed by the condensation of the shell and, with it, the establishment of a biphasic state (Figure 1; Wheeler et al., 2017; Niewidok et al., 2018). SG disassembly is a reverse process that starts with shell diffusion, next by core dissipation (Figure 1G; Wheeler et al., 2017). This section will discuss the accumulated knowledge on SG assembly in mammals and yeast, highlighting the available evidence in plants to give the reader a general overview of the existing gaps between non-plant and plant models and the potential research fields that remain to be addressed.

## Nucleation Events

**FIGURE 2:**
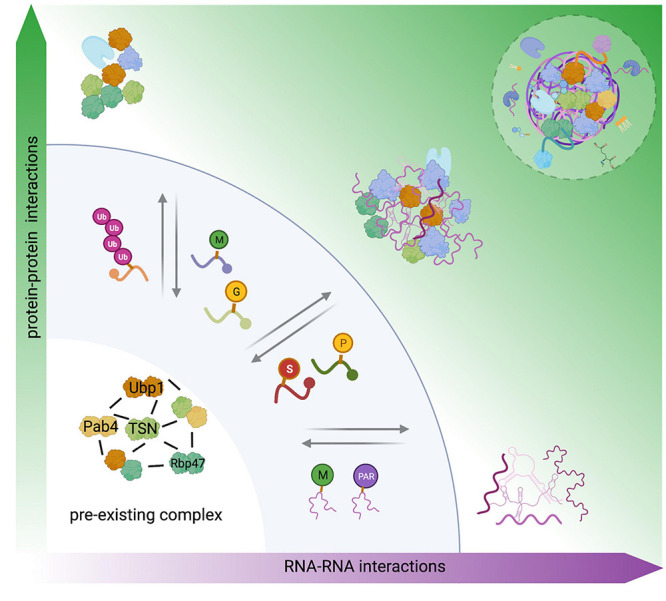
Role of PTMs in the four-phase SG assembles model. SG nucleation assembly is described on the function of concentration and combination of either protein–protein, RNA–RNA interactions, or protein–RNA interactions. The evidence indicates the presence of preexisting complex under non-stress conditions. In line with this finding, we suggested this complex as a steady–ready interaction in the first step in the four-phase model, where PTMs and RNA modifications might function as a signal to regulate oligomerization, structure, subcellular localization, and protein functions, these evoked changes on SG components could modulate SG assembly or disassembly, independent of concentration. Modified from Van Treeck and Parker (2018). Created with BioRender.com.

High local concentrations of mRNP complexes can trigger the first step in SG nucleation (Protter and Parker, 2016). However, nucleation is also a stepwise process modulated by the valence (the number of available interaction domains) encoded in core proteins and RNA molecules, RNA influx, competitive protein–protein interactions (PPIs), RNA–RNA, and RNA–protein interactions (Ditlev et al., 2018; Sanders et al., 2020). Additionally, PTMs, such as protein methylation, phosphorylation, glycosylation, hypusination, ubiquitination, and nucleic acid modifications (PARylation and methylation), comprise an extra level of regulation that can negatively or positively influence the nucleation events [Figure 2; reviewed in Protter and Parker (2016)].

Four types of PPI are involved in SG dynamics: stereo–specific interactions between well-folded domains, the interactions of short linear motifs (SLiMs) and well-folded domains, specific interactions between local structures IDR (e.g., LARKs), and promiscuous interactions between IDRs (pi–pi, cation–pi, and charge–charge) (Hurtley, 2018; Van Treeck et al., 2018). Promiscuous IDR interactions are probably the drivers in the first steps of nucleation. Once the concentration threshold is reached, these forces can develop into more stable and stronger interactions (Van Treeck et al., 2018).

Remarkably, RNA plays one of the most crucial roles in the SG nucleation process. RNA interactions by themselves promote LLPS and drive SG assembly *in vitro* through Watson–Crick base-pairing, non-canonical base-pairing, and helical stacking (Van Treeck and Parker, 2018; Van Treeck et al., 2018). Indeed, under stress conditions, RNA–RNA interactions are favored (Maharana et al., 2018; Van Treeck et al., 2018). Consistently, Maharana et al. (2018) and Tian et al. (2020) reported RNA–RNA networks as crucial SG components, which were able to fine tune nucleation, stability, morphology, the selective control of SG transcriptome and proteome, and therefore the SG function. In summary, SG nucleation results from a series of events that favor the synergistic interactions of a required pool of RNA, proteins, or both.

## RNA and Protein Interactions: The Driving Forces of Core Growth

So far, the known mammalian, yeast, and plant SG core proteins that drive LLPS display a multi-domain architecture, usually encode oligomerization domains (ODs), RNA-binding domains (RBDs), IDRs, low-affinity arginine-rich motifs (RGG), and PrLD (Sanders et al., 2020). The modular architecture was first described in mammalian SG core proteins, such as well-characterized G3BP1, UBAP2L, and TIA-1 proteins (Jain et al., 2016; Protter and Parker, 2016; Huang et al., 2020). In plants, the Rbp47 and Rbp45 are the canonical examples of this modular architecture in SG core proteomes. The accumulated SG proteomic evidence suggests the multi-domain architecture as an essential trait of SG core proteins in non-plant and plant models. In agreement, the Rbp47b interactome data of *A. thaliana* SGs revealed conserved SG core proteins enriched in PrLD, ATPase, and RBDs (Kosmacz et al., 2019). The study of specific plant proteins encoding non-canonical RBDs might unveil the unknown roles of these proteins in SG assembly and function (Lorković and Barta, 2002; Lorković, 2009).

In addition to the multi-domain architecture, the determinant factors that trigger the core growth process are the hetero- and homo-oligomeric interactions between the nucleation proteins of SG (Huang et al., 2020). SGs-PPI networks physically increase the RNA-binding surface of the reported mammalian G3BP1 and G3BP/UBA2PL oligomers (Guillén-Boixet et al., 2020; Huang et al., 2020) by acting as recruiting scaffolds for mRNA and other SG nucleation proteins. Interestingly, protein interactions can also prevent SG core growth, as reported by the interaction between USP10/G3BP in mammals (Kedersha et al., 2016). In plants, Bhasin and Hülskamp (2017) propose the AN gene in *A. thaliana* as a negative regulator of plant SG assembly. The interactome screening of the AN protein revealed a direct association with SG core proteins. The increased SG density in *AN* mutants suggests AN interactions as a negative modulator of SG formation (Bhasin and Hülskamp, 2017).

In contrast to mammals and yeast, the oligomerization of SG core proteins in plants during the nucleation process has been poorly studied. However, Kosmacz et al. (2018) reported the *in vitro* Rbp47b self-oligomerization in the presence of the small molecule 2′,3′-cAMP suggesting a role as a facilitator of SG assembly. In plants, the function of small molecules such as Zn^2+^ in SG assembly is not well understood. Nonetheless, Zn^2+^ is an integral component of the membranes and cofactors of enzymes and hormones, being crucial in plant nucleic acid metabolism that might be involved during plant SG assembly. However, the role of small molecules in plant SG dynamics is an appealing field of investigation (Umair Hassan et al., 2020).

Of particular interest is the RNA secondary structure during the core growth process, which can act as a determinant factor for its tagging into liquid condensates. The mRNA-specific configurations can selectively expose or mask complementary sequences to interact with other RNAs, determining the formation of homotypic and heterotypic RNA complexes within SGs (Langdon et al., 2018; Tian et al., 2020). Supporting *in vitro* evidence provided by Langdon et al. (2018) in the filamentous fungus *Ashbya gossypii* suggests that the presence of the RNA-binding protein Whi3 induces a structural change in the cyclin CLN3, formin (actin) BN1, and SPA2 transcripts, leading to the establishment of homotypic CLN3 and BN1/SPA2 heterotypic mRNA complexes (Langdon et al., 2018; Tian et al., 2020). These mRNA complexes showed selective behavior, excluding the mutually present components. The authors proposed that the induced secondary structure in CLN3 and BN1 and SPA2 mRNAs by Whi3 protein determined the recruited mRNA identity in SGs and the assembly of distinct granules in a single cell (Langdon et al., 2018; Tian et al., 2020).

In line with mRNA structural studies, De Groot et al. (2019) demonstrated a positive correlation between highly structured RNAs and a protein-binding affinity; these hybrid interactions guide the assembly and determine the composition of the RNP granules in humans. The SG transcriptome and the role of the RNA structure are less known in plants, and only a few studies have addressed the composition of plant SGs. GO ontology analyses suggest an enrichment of structured transcripts in the regulatory processes that require highly interconnected protein networks, such as stress responses (Deng et al., 2018). Nonetheless, the role of RNA structures in SG nucleation still awaits investigation.

The accumulated data in non-plant models showed that RNA pools promote the RNA networks that serve as scaffolds for RBPs and promote SG core growth through RNAs–RBPs interactions (Bounedjah et al., 2014; Protter and Parker, 2016; Van Treeck et al., 2018). Such a mechanism has been reported to regulate *A. thaliana* hypoxia response. Under non-stress conditions, UBP1C interacts with uracil-rich 3′ UTRs mRNAs. During hypoxia response, after polysome disassembly and cytoplasmic RNA influx, promiscuous UBP1C-mRNA binding resulted in SG assembly (Sorenson and Bailey-Serres, 2014). Nevertheless, not all RNA–RBP interactions favor the formation of SGs as demonstrated in *in vitro* and mammalian cells. Contrary to the previous example, the DEAD-box protein eIF4A (an ATP-dependent RNA helicase), a well-known conserved component of SGs, and other RNP granules across eukaryotes can negatively regulate SG formation, limiting RNA condensation in *in vitro* and in mammalian cells (Tauber et al., 2020). In this regard, eIF4A-overexpressing cells resulted in defective SG formation, while an eIF4A-RNA-binding mutant showed no SG inhibition. *In vitro*, recombinant eIF4A1 abolished RNA condensation in an ATP-dependent manner, suggesting that the inhibition effect was driven by a direct eIF4A–RNA interaction (Tauber et al., 2020). So far, in plant models, there is no evidence of the RNA–RBP interactions that negatively affect SG formation.

## An Evolutionary Conserved Preexisting Complex Across Eukaryotes

The evidence from mammals, yeast, and plants has revealed a preexisting or steady–ready interaction network between the core SG proteins, even in unstressed cells. These networks are suggested to boost the stress response and enable a rapid coalescence into larger SGs upon cell exposure to challenging conditions (Markmiller et al., 2018; Youn et al., 2018; Kosmacz et al., 2019; Gutierrez-Beltran et al., 2020). In plants, Rbp47b, UBP1C, and TSN are the *bona fide* SG core components of this steady–ready complex (Sorenson and Bailey-Serres, 2014; Kosmacz et al., 2019; Gutierrez-Beltran et al., 2020). This finding raises an interesting question of how cells control the behavior of these preexisting complexes. In mammals, yeast, and insects, PTMs of core RBPs (e.g., glycosylation, phosphorylation, and ubiquitination) and RNA modifications (methylation) act as cellular switches to activate or suppress SG nucleation. These modifications influence protein and RNA structures, oligomeric states, enzymatic activities, and intracellular locations (Kyung Lee, 2012; Protter and Parker, 2016; Dao et al., 2018; Ries et al., 2019; Söding et al., 2020). In this regard, Sorenson and Bailey-Serres (2014) opened up the discussion about the possibility for the putative phosphorylation of the UBP1C protein to activate its aggregation into SGs during hypoxia. The homology of core proteins (RBPs) in eukaryotes might suggest a similar active regulation of plant SG nucleation. Nonetheless, this unexplored field requires further investigation.

Finally, SG assembly is summarized in the previously reported “four-phase” model (Van Treeck and Parker, 2018). Here, protein–protein, RNA–RNA, and protein–RNA interactions are the three key factors to drive SG nucleation. The fourth factor comprises competing interactions of RNA–RNA or RNA–RBPs interactions, and RBP depletion is a common mechanism used by cells to limit and regulate SG assembly (Figure 2). We propose the preexisting complex as the first step in the “four-phase” model, where RNA modifications and PTMs might act as SG on/off switches, playing an essential role in this model (Figure 2).

## Shell Assembly: Growth and Fusion Phase

Structurally, SGs are biphasic assemblies composed of several dense cores of ∼0.2 μm in diameter immersed in a less concentrated and dynamic shell of variable size (Youn et al., 2019). Shell assembly is described as a two-phase process: the growth phase and fusion phase (Wheeler et al., 2016; Figure 1E). Once the SG core is established, the SG core RBPs recruit additional proteins *via* a high local concentration of IDRs that promote LLPS and allow shell growth, forming microscale SGs. Usually, these newly added proteins are often shell-specific proteins with no RNA-binding activity but stress, cell type, or organism dependent (Markmiller et al., 2018; Riggs et al., 2020). The second phase involves the assembly of higher-order structures by the fusion among several microscale SGs to eventually establish a multicore SG structure up to ∼2 μm in size (Lin et al., 2015; Wheeler et al., 2016; Youn et al., 2019; Riggs et al., 2020).

The core and shell are found to differ in the compositional, dynamic, and functional levels across eukaryotes (Jain et al., 2016; Markmiller et al., 2018; Gutierrez-Beltran et al., 2020), whereas individual cores reportedly form stable structures of an average size of 0.25 μm over time due to a specific and stable PPI. Transient and weak interactions among the IDRs present in the shell make this phase very dynamic (Protter and Parker, 2016; Wheeler et al., 2016). It is important to emphasize that stable protein interactions might also be present in the shell fraction, but requires further investigation in all studied models.

Mammalian and yeast experiments have consistently shown that SG assembly/disassembly is not a passive mechanism but requires energy (Jain et al., 2016; Shao et al., 2017). ATP-depletion assays in non-plant models have shown a crucial role of ATP-dependent remodeling complexes in SG assembly and dynamics. However, this role might change across groups and needs to be investigated in each particular case (Chantarachot et al., 2020). Despite this, the contribution of ATP during SG plant assembly is not clear. Metabolomic analysis using Rbp47b marker lines unveiled the presence of ADP and proteins with ATPase activity in *A. thaliana* lysates, which suggests the presence of ATP in plant SGs (Kosmacz et al., 2019). Whether ATPases are active or not inside plant SGs, and the precise functions of ATP need further investigation.

To date, the best-characterized SGs plant proteins are of the *A. thaliana* TSN proteins, the evidence have suggested a crucial role of these proteins in SG assembly, stability, and identity (Gutierrez-Beltran et al., 2020). The TSN interactome data disclosed an enrichment of ATP-dependent remodeling complexes, such as chaperonin-containing T complex, or DEAD-box RNA/DNA helicases in the TSN stress-sensitive fraction. Using a co-immunoprecipitation (co-IP) approach, Gutierrez-Beltran et al. (2020) demonstrated a direct TSN2 interaction with at least one component of the DEAD-box ATP-dependent RNA helicase family (RH12). ATP-dependent remodeling enzymes interact with the TSN preexisting complex and might play a role in the early stages of SG assembly. Upon stress perception, the CCT or DEAD-box RNA/DNA helicases are released from the preexisting complex to favor shell assembly (Gutierrez-Beltran et al., 2020). In line with these data, the CTT complex from yeast negatively regulates SG assembly with no impact on SG clearance (Jain et al., 2016).

Interestingly, knockout TSN mutant lines showed a delayed SG assembly, accompanied by a noticeable reduction of the SG Rbp47b foci probably due to an accelerated exchange rate of Rbp47b, as observed by fluorescence recovery after photobleaching (FRAP) studies. By contrast, the fluorescent signals of TSN1- and TSN2-labeled proteins in over-expression lines could not be recovered after bleaching treatment. The deletion of the TSN gene SGs allowed the co-localization of the eIF4E with PB, which was usually not present in these structures. Collectively, these data suggest that (1) TSN proteins are part of a stable SG core fraction, (2) they serve as a scaffold platform for other core components during the assembly process, and (3) they are the crucial regulators of SG identity (Gutierrez-Beltran et al., 2015, 2020).

The assembly of SGs is mainly driven by the LLPS process. However, time-lapse analyses have revealed a well-orchestrated motor-driven transport of microscale SG cores in plant and non-plant models (Gutierrez-Beltran et al., 2015; Hamada et al., 2018; Perez-Pepe et al., 2018). Briefly, *A. thaliana* transgenic lines co-expressing RFP-TSN1/2 and GFP-Rbp47b constructs showed that TSN1–2 and Rbp47b proteins were transported in a microtubule (MT)-dependent way. The treatment with either amiprophos-methyl (APM) and a MT depolymerization promoter or with taxol, an MT stabilizer showed a reduction in TSN granule density, suggesting that MT polymerization and depolymerization were equally critical to SG assembly (Gutierrez-Beltran et al., 2015). Similarly, Hamada et al. (2018) used a mixed drug treatment with a MT polymerization inhibitor, oryzalin, and latrunculin B, an actin polymerization inhibitor, to show that, in *A. thaliana*, lines expressed the eIF4A2-GFP construct. The disruption of these cytoskeleton components blocked the fusion phase and long-distance transport of SGs. Altogether, these results suggest that polymerization is critical in the early steps of plant SG formation. Meanwhile, depolymerization seems to be relevant in the fusion phase. By contrast, actin filaments are dominant in a long-distant SG transport (Gutierrez-Beltran et al., 2015; Hamada et al., 2018).

## SG Disassembly: Shell Dissipation and Core Dissolution

The equilibrium between SG assembly/disassembly is essential under stress response and growth resumption upon stress recovery (Hu et al., 2017). SG disassembly is as strictly relevant as its assembly. Meanwhile, the knowledge of SG formation has increased over time, there is still a long way to go in understanding the compositional changes, molecular signals, and PTMs that govern SG disassembly in eukaryotes (Wheeler et al., 2016). In yeast and mammals, the SG disassembly is a reverse ordered process that starts with shell dissipation followed by core dissolution (Figure 1G; Wheeler et al., 2016; Perez-Pepe et al., 2018). The SG shell is proposed to assemble through weak interactions. In plants, Chodasiewicz et al. (2020) observed that the treatment with 1,6-hexanediol (which interferes with weak hydrophobic PPI and protein–RNA interactions) decreased the size of SCO1-GFP foci in cpSGs by around 30%. In this regard, perturbations that weaken the interactions between IDR might be enough to drive shell dissipation in plant and non-plant models (Wheeler et al., 2016; Perez-Pepe et al., 2018).

Furthermore, human evidence suggests that ubiquitination might assist shell disassembly. UBQLN2 is a proteasome adaptor protein that recognizes Ub molecules on substrate proteins and directs them to degradation. UBQLN2 *in vitro* can drive LLPS; *in vivo*, the UBQLN2 protein is co-localized to SGs. Interestingly, the addition of ubiquitin or poly-ubiquitin chains promoted SG dissolution. The authors suggested that this tagged recognition mechanism may help shuttle proteins out of the SGs (Dao et al., 2018). In plants, the interactome analysis of Rbp47b revealed an ovarian tumor domain (OTU)- containing dub (deubiquitinating enzyme) 2 (OTU2) as a component of SGs (Kosmacz et al., 2019). However, further assays are required to clarify its function in plant SG formation.

In mammals and yeast, core dissolution requires ATP-dependent remodeling complexes, such as chaperones, helicases, and cytoskeleton components, to clear stable protein associations (Van Treeck and Parker, 2018). Marmor-Kollet et al. (2020) reported that the SG dissociation process in mammalian cells involves sHSPs, RNA helicases, cytoskeletal proteins, and the additional recruitment of disassembly engaged proteins (DEPs), which are related to autophagy and ubiquitin pathways. The authors also demonstrated the SUMOylation of SG proteins as a requirement for its disaggregation (Figure 1G; Marmor-Kollet et al., 2020). Recently, Maxwell et al. (2021) reported the essential role of ubiquitination for the fast disassembly and cell recovery of SGs after heat stress. The reversible mRNP remodeling activity, nucleo-cytoplasmic transport, and the resumption of the translation process were principally affected by impaired ubiquitination in human cells. G3BP1 was interestingly found to be ubiquitinated during heat stress but not under other tested treatments. In a complementary study performed by the same group of scientists, it was demonstrated that ubiquitination is fundamental for a proper SG disassembly. The presented evidence showed that ubiquitinated-G3BP1 interacts with valosin-containing protein (VCP), an ubiquitin-dependent protein segregase, promoting the G3BP1 extraction during SG disassembly. In this sense, G3BP1 versions not able to be ubiquitinated showed an impaired interaction with VCP and aberrant behavior during disassembly (Gwon et al., 2021).

In line with these findings, in plants during the heat-recovery phase of *A. thaliana*, the heat shock HSP70 and HSP101 proteins are re-localized to SGs to promote core dissolution; as recovery proceed, the HSP70 and HSP101 proteins are redistributed in the cytoplasm. Knockout *hsp101* mutant was affected in SG dissociation after stress (Cherkasov et al., 2013; Merret et al., 2017; McLoughlin et al., 2019). Affinity isolation of *A. thaliana* protein extracts followed by mass spectrometry analysis had helped to elucidate the molecular function of these chaperones during heat stress and subsequent recovery. HSP101 interacts transiently with class I and II sHSP, HSP70, and the proteasome regulatory particle subunit RPN1. Because the deficiency of HSP101 or class I sHSPs proteins increased the proportion of ubiquitylated proteins during heat stress, the authors suggested that the interaction of HSP101-proteasome units served as a protein triage center to avoid the proteotoxic stress caused by protein aggregation of those proteins that failed to disaggregate after heat stress (McLoughlin et al., 2019).

The convergence of these findings in plants and mammals suggests an evolutionarily conserved process in eukaryotes during the involvement of sHSPs, HSPs, and protein degradation in the disassembly of SG. However, the role of SUMOylation and DEPS in SG disassembly has not been studied yet in plant models.

Interestingly, the screening of a library composed of natural compounds isolated from traditional Chinese medical plants to find the molecules influencing SG dynamics in human HeLa cells expressing a TIA-1-GFP construct revealed two plant benzene derivates, syringic acid, and troxerutin, to promote TIA-1-SG disassembly. Both compounds are specific modulators of SG dissociation and do not affect other cytoplasmic granules. In particular, syringic acid facilitates cell stress recovery (Hu et al., 2017). In plants, the screening of molecules with regulatory activity remains to be addressed.

## Relevance of SGs in Plant Stress Resilience

Stress granules are implicated in many disease pathologies in humans, including amyotrophic lateral sclerosis, Alzheimer’s disease, and antiviral responses (Wolozin and Ivanov, 2019; Eiermann et al., 2020). In this sense, SGs have also been shown to protect cells from apoptosis, reducing the number of reactive oxygen species (Takahashi et al., 2013; Thedieck et al., 2013), and may also regulate viral replication upon infection (Beckham and Parker, 2008; Eiermann et al., 2020). SGs assemble when eukaryotic cells are exposed to injuries and quickly dissipate after stress removal. This process is variable in time with an average of 15 min to 1 h, and it is dependent on the nature of stress, dose, and exposure time (Chantarachot and Bailey-Serres, 2018). Notably, abnormal aggregation or persistence of SGs can be deadly to cells (Mann et al., 2019).

Numerous environmental stresses trigger plant SG assembly, including high salt, heat, darkness, hypoxia, the inhibition of oxidative phosphorylation, and viral infection (Weber et al., 2008; Sorenson and Bailey-Serres, 2014; Yan et al., 2014; Gutierrez-Beltran et al., 2015). Rbp47b and UBP1B proteins are required for SG formation in *A. thaliana* plants under heat stress (Weber et al., 2008). The overexpression of the *UBP1B* gene leads to an increased number of SGs and a heat stress-tolerant phenotype, whereas the *ubp1b* mutant plants were more sensitive to heat, salt, and osmotic stress (McCue et al., 2012; Nguyen et al., 2016). Moreover, UBP1B is required for a plant response to abscisic acid (Nguyen et al., 2017). Similarly, the impaired expression of the *UBP1C* homolog gene affected plant survival under hypoxia stress. Plants-overexpressing *UBP1C* fused to GFP displayed oxygen-regulated granule formation (Sorenson and Bailey-Serres, 2014; Table 1).

Many TSN1-SGs are formed in the cytoplasm of transgenic *A. thaliana* plants in response to salt stress. The overexpression of TSN1 led to increased salt stress tolerance. Plants with *tsn* deficiency showed a stress-sensitive phenotype to salt and heat stress (Gutierrez-Beltran et al., 2015). Further, TSN positively regulates the transcript levels of the *GA20ox3* gene, a key enzyme for GA biosynthesis. Thus, the overexpression of *TSN1* resembles the phenotypes related to the overproduction of GA while *tsn*-deficient mutant lines showed a slower growth response under salt stress similar to *ga20ox3* mutant plants (Yan et al., 2014).

*Arabidopsis thaliana* mutants that were impaired in *AN* gene expression showed a high number and reduced size of SGs under heat stress; similarly, the *AN* mutant plants were tolerant to osmotic and salt stress, in contrast to the wild type. AN may act as a negative regulator of stress responses (Bhasin and Hülskamp, 2017).

AtG3BP-2 (according to Reuper et al., 2021) is an RNA-binding protein homolog of human G3BP1, which are localized to plant SGs and might play a role in plant virus resistance. Like mammals, plant virus proteins bind to AtG3BP-2 and probably inhibit the formation of SGs (Krapp et al., 2017). Further studies involving virus infection under real conditions will help to confirm this hypothesis. Besides, *A. thaliana* HSP101 is an indispensable chaperone for plants surviving extreme heat stress. HSP101 protein is remarkably recruited into SG during heat stress and remains in the aggregates under recovery (Merret et al., 2017; McLoughlin et al., 2019).

The regulation of mRNA dynamics is essential for growth, development, and stress responses. The RNA DHH1/DDX6 helicases of *A. thaliana* RH6, RH8, and RH12 show a redundant functional role in mRNA decay, which is essential for proper growth and plant development. RH6, RH8, and RH12 associated with PBs and SGs contribute to the assembly of these foci (Chantarachot et al., 2020). The number of hypoxia-induced SGs was reduced in the *rh6812* triple mutant, and the accumulation of mRNAs was related to defense responses. Elevated levels of the phytohormone salicylic acid were also detected. In this regard, mutant plants displayed constitutive defense responses and were more resistant to pathogen infections (Chantarachot et al., 2020).

Jung et al. (2020) indicated that the *A. thaliana* transcription factor ELF3 plays a crucial role during heat temperature responses. ELF3 contains a polyQ stretch within a predicted prion domain (PrD), which allows the formation of droplets in response to high temperatures. The authors hypothesize that these stretches modify the solubility of ELF3 protein. The homolog of ELF3 proteins that do not contain detectable PrD failed to revert the temperature-sensitive phenotype of elf3 mutant plants. Comparative sequence studies among plant species show a correlation of long polyQ tracks with the temperature responsiveness. Notably, SG core is enriched in PrD proteins.

In summary, SG formation is an early response to stress, and the mutant plants impaired in SG assembly or disassembly show abnormal responses under the circumstances of stress, which suggest the importance of a coordinated action of SG to promote stress tolerance.

## Role of SGs as Modulators of Cell Signaling and Metabolism

The most accepted model suggests that SGs may act as a triage center of mRNA, involved in sorting, remodeling, and exporting specific mRNAs for reinitiation, decay, or storage. SGs may also protect the proteins from unfolding or degradation (Anderson and Kedersha, 2006; Vanderweyde et al., 2013; Protter and Parker, 2016; Chantarachot and Bailey-Serres, 2018). Various pieces of evidence have suggested alternative roles for SGs. Takahara and Maeda (2012) reported the sequestration of the target of rapamycin complex 1 (TORC1), a principal regulator of the cell cycle (Loewith, 2010), into SGs under heat stress conditions, which allows a direct coordination of the reactivation of TORC1 through SG disassembly in the recovery phase.

In plants, Gutierrez-Beltran et al. (2020) found that SnRK1, a primary metabolic sensor (Crozet et al., 2014), is targeted to SGs exclusively during heat stress. Its activation is dependent on the formation of heat-induced SGs and its interaction with TSN2, an integral component of SGs (Gutierrez-Beltran et al., 2015). Kosmacz et al. (2019) observed that CDKA 1, a central cell-cycle regulator (Dissmeyer et al., 2007), in *A. thaliana* is localized into SGs under heat stress conditions. Finally, chloroplast SGs sequestrate the factors required for photosynthetic activity. For instance, mRNA encoding for a large subunit of Rubisco is recruited inside alga chSGs under high light stress (Uniacke and Zerges, 2008). Moreover, Rubisco activase and Rubisco accumulation factors were also present in heat-induced chSGs from *A. thaliana* (Chodasiewicz et al., 2020). cpSGs may recruit key photosynthetic proteins as a mechanism to protect them from stress or temporarily deactivate them as regulatory constituents, which are important for plant growth and stress responses (Chodasiewicz et al., 2020).

Considering the relevance of modulating cell signaling and metabolic pathways, there is a putative novel role for SGs in which the recruitment of relevant enzymes to SGs provides a multifaced mechanism for a rapid regulation in response to environmental conditions.

## Contrasting Evidence of the Role of SGs

The role of SGs has remained controversial since the beginning of its study. Using FRAP in human cells, Mollet et al. (2008) showed that the residence time of mRNP complexes in sodium arsenite-induced SGs was in the range of ∼1 min. However, SGs were present for up to 3 h. This transient entry of mRNA was not due to degradation but to a rapid and dynamic exchange of mRNPs between the SGs and the cytoplasm. These observations argued against the role of SGs in targeting mRNA for degradation or mRNP storage under stress. Furthermore, these conclusions were complemented by remarks made in human cell lines by different authors. Souquere et al. (2009), by using *in situ* hybridization studies, reported that poly(A)^+^ mRNAs in SGs represent just a minor portion (∼15%) of the cellular mRNA. Supporting evidence was reported by Sheinberger and Shav-Tal (2017) after studying the localization of different mRNAs by employing MS2-tagged mRNAs and FISH analyses. Khong et al. (2017) also concluded that about 10% of the total mRNA in the cell accumulates in SGs, based on the quantification of the RNAseq of isolated SG cores and oligo(dT) FISH. These data suggest that SGs do not work as storage sites under stress.

Further, G3BP-deficient cells (Tourrière et al., 2003; Matsuki et al., 2013) can repress global translation without forming SGs (Kedersha et al., 2016), suggesting that SGs do not have a significant influence on global translation (Mateju et al., 2020). Khong et al. (2017) noted that there is no excessive abundance of specific mRNA within mammalian SGs as mRNA from almost every expressed gene is partially present in SGs. However, none of them represents more than 1% of the total RNA SG molecules, suggesting that the interactions required for SG localization are generic and not limited to a specific subset of mRNA.

Khong et al. (2017) and Mateju et al. (2020) found that, in mammals, non-translating mRNAs are more susceptible to be localized to SGs. Meanwhile, new evidence has revealed that some SG can recruit 60S subunits and undergo translation Moon et al. (2019), Mateju et al. (2020) suggest that mRNAs can transiently interact with SGs when still being attached to ribosomes. Mateju et al. (2020) observed a high cell-to-cell variability with an average of 30% of SG-localized reporter mRNAs undergoing a translation, which would mean that it is not a rare event. On the other hand, 98% of lysine demethylase 5 B (KDM5B) SG-associated reporter mRNAs employed by Moon et al. (2019) were not translated, which supports the hypothesis that translation repression is a general requirement for localization to SGs. However, it is not opposite to the findings of Mateju et al. (2020) as Khong et al. (2017) reported that some particular mRNA species could localize up to 95% of their total cellular bulk into SGs. Nevertheless, mRNA from almost every expressed gene is partially present in SGs, but none of them represent more than 1% of the total SG RNA molecules, suggesting that the interactions required for SG localization are generic and not limited to a specific subset of mRNA.

Both authors agree that most SG-localized mRNAs are stalled with preinitiation complexes. However, the rate of SG-localized mRNAs undergoing a translation remains unclear, which may affect the precept of whether SG-associated mRNAs are being translated or not. Taken together, these observations lead to the conclusion that previously reported slight overrepresentation of non-translating mRNAs in SGs would be better explained by the fact that non-translating mRNAs are preferentially recruited to SGs, instead of the repression of the translation as a direct consequence of sequestration into SGs. Indeed, SGs do not directly repress, at least in human cells, any aspect of mRNA translation (Mateju et al., 2020). In this regard, Wilbertz et al. (2019) showed that mRNAs sequestered into SGs under stress in the recovery phase are translated with the same efficiency as those mRNAs that remained outside. Therefore, it seems that mRNA localization to SGs also has no effect on translational capacity under stress or in the recovery period.

Interestingly, Khong et al. (2017) noticed that not only non-translating mRNAs but also longer transcripts are preferentially recruited to SGs. This observation was confirmed by Mateju et al. (2020) and further extended by Moon et al. (2019), where the authors described that mRNAs have frequent transient interactions with SGs but can occasionally enter and establish lasting associations. These associations were also favored in larger SGs, showing stronger interactions with mRNAs compared to smaller SGs. A hypothesis suggested by Van Treeck et al. (2018) is that long mRNAs accumulate in SGs due to more non-specific *trans* RNA–RNA interactions. Indeed, it would be reinforced by the fact that one end of the mRNA can extend beyond the boundaries delimited by the protein components, putatively providing an extended interaction surface that could promote the fusion of smaller SGs or docking SGs and PBs (Moon et al., 2019). Hamada et al. (2018) also previously observed the same in *A. thaliana* cells.

In humans, the cytosolic m^6^A-binding proteins YTHDF1, YTHDF2, and YTHDF3 are relocalized to SGs under different stimuli and in a range of cell types (Ries et al., 2019). After measuring the m^6^A levels in mRNA purified from SGs, Ries et al. (2019) found that the levels of m^6^A were about 50% higher than those in the total cellular mRNA. In addition, they also observed that the number of m6A sites is correlated with the SG enrichment of mRNAs, even when the length is similar, thus adding another variable in the mRNA that favors its localization in SGs.

Stress granules have been defined as the assemblies of untranslated mRNPs that are formed from mRNAs stalled in translation initiation (Protter and Parker, 2016; Khong et al., 2017), which has been considered as a fundamental property of SGs. Mateju et al. (2020) studied the relationship between mRNA localization and translation under stress by single-molecule imaging techniques for mRNA (particularly MCP-Halo and SunTag). MS2 tagging system allows the visualization of individual reporter mRNAs, whereas SunTag concedes the simultaneous visualization of nascent peptide chains. Contrastingly, they observed that mRNAs (regardless of whether their translation is enhanced or inhibited under stress) localized to SGs can undergo a complete translation cycle and even can be transported between cytosol and SGs without modifying their translational status, arguing against a direct role for SGs in the inhibition of protein synthesis. The authors concluded that the previously reported assumptions on the contribution of SGs in translation repression and their composition based on non-translating mRNAs were founded on the observation that SG formation coincides with a global silencing of translation but were not supported by a direct observation.

There is still no evidence to confirm whether plants possess the dynamics of SG-associated RNAs are similar to those reported in mammals and yeast or whether they show their singularities. Transcriptomic analyses in combination with single-molecule imaging approaches will certainly contribute to the clarification of the status and fate of the RNAs sequestered to SGs under stress. Further analyses in plants and yeast involving a wide variety of mRNA reporters with different *cis*-elements would clarify some still controversial issues. For this, establishing single-molecule imaging platforms suitable for each organism is critical, particularly for the direct observation of the SG-associated mRNA dynamic prior to, during, and after different stress stimuli.

## Relationship Between Sgs and Other Cytoplasmic Foci

Stress granules may coexist in eukaryotic cells with other types of dynamic cytoplasmic mRNP complexes assemblies called PBs. PBs are physically, compositionally, and functionally associated with SGs in eukaryotes (Figure 2; Buchan and Parker, 2009; Chantarachot and Bailey-Serres, 2018). Although it is believed that PBs are involved in mRNA decay (Sheth and Parker, 2003; Kedersha et al., 2005; Kedersha and Anderson, 2007), it has been shown that mRNAs targeted into PBs can be stabilized instead of degraded, even though several mRNA decay factors might be found among the key components of PBs (Chantarachot and Bailey-Serres, 2018). There are many doubts and confusion regarding the function of PBs in cells and in mRNA regulation. Weber et al. (2008) verified the existence of PBs in plants and showed that they are similar to those previously described in humans (Kedersha et al., 1999) and yeast (Sheth and Parker, 2003).

Intrigued by the observation that arsenite-induced SGs appeared to be juxtaposed with PBs, Kedersha et al. (2005) addressed for the first time the relationship between SGs and PBs. A possible physical SG–PB interaction was further studied under a microscope using different fluorescent-tagged SG- or PB-specific protein markers. Researchers have reported that SGs are often associated with one or more PBs. Meanwhile, some PBs may remain bound to SGs over time, and the remaining PBs can move freely in the cytoplasm with no interaction with SGs. Interestingly, they also noted that the overexpression of tristetraprolin (TTP, also known as ZFP36), a protein involved in mRNA decay and associated with SGs or its close homolog butyrate response factor 1 (BRF1, also known as ZFP36L1), can stabilize the interaction between SGs and PBs, increasing both the number and duration of the interactions (Kedersha et al., 2005).

Tristetraprolin belongs to the group of human tandem CCCH-type zinc finger proteins and possesses RNA-binding activity conferred by zinc finger motifs. It has also been proposed that TTP nucleates PB formation in human cells (Franks and Lykke-Andersen, 2007). In *A. thaliana*, some TZF CCCH-containing TZF protein family members reportedly colocalize with SGs and PBs (Maldonado-Bonilla, 2014; Bogamuwa and Jang, 2016). However, no orthologs have been identified, and it has not been demonstrated so far whether some of the *A. thaliana* TZF proteins could play a role similar to human TTP. Weber et al. (2008) later confirmed the results obtained by Kedersha et al. (2005) using a similar strategy but in tobacco mesophyll protoplast. Using co-localization studies of different marker proteins for SGs as well as for PBs, the authors revealed not only the proximity of SGs and PBs but also of SGs and HSGs.

Using several *Saccharomyces cerevisiae*-mutated strains displaying the defects in PB assembly, Buchan et al. (2008) demonstrated that SG assembly is also affected, indicating the dependency of SGs on PB formation. However, PB formation is independent of SG formation. This may suggest that mRNA moves from PBs to SGs, and SG formation is stabilized by a preexisting pool of mRNP in PBs. These conclusions were made according to an extension of the work of Hondele et al. (2019), where the authors showed that RNA-dependent DEAD-box ATPases (DDXs) regulate the RNA release or transfer between phase-separated organelles. Further evidence was provided by the use of yeast strains lacking DHH1 (the ortholog of DDX6 in humans), which cannot efficiently form PBs, or expressing its ATPase-deficient variant (and therefore forming non-dynamic PBs) in addition to a subsequent *in vitro* reconstitution of the process. The authors observed that the ATPase activity of this enzyme is essential for regulating the flux of mRNAs between PBs and SGs, and therefore, also affecting the assembly of SGs (Hondele et al., 2019). Similar to Buchan et al. (2008), these observations indicate that, at least in yeast, mRNA has to pass through SGs and PBs before they can contribute to the assembly of SGs. However, this hypothesis remains to be tested in plants.

Further observations made by Chantarachot et al. (2020) regarding the study of DHH1/DDX6-like proteins in *A. thaliana* showed that under stress, RHs overlap not only with SG but also with PB markers, and that the triple *rh6812* mutants displayed a temporarily limited increased number of SGs and PBs. Neither the SG nor the PB assembly was totally suppressed. It seems that even when SGs are evolutionarily conserved, each lineage may present its singularities, as, for example, the depletion of DDX6 in human cells does not affect SG assembly (Serman et al., 2007). In fact, Wilbertz et al. (2019) could not detect the transit of mRNAs from PBs to SGs in human cells, whereas the observed movement of mRNAs from SGs to PBs was quite unexpected. This indicates that SGs are unlikely to correspond to an mRNA triage center based on the previously observed SG and PB proximity.

Hamada et al. (2018) addressed the relationship between SGs and PBs in plants by performing time-lapse observations through high-resolution high-sensitivity confocal microscopy. The authors found that SGs can fuse with other SGs associated with PBs; however, SGs did not perfectly overlap with PBs, and the size of SGs did not correlate with the size of PBs. Moreover, once SGs were associated with PBs, they did not dissociate. Furthermore, upon extended incubation periods, the fusion of SGs progressed, but most of the PBs were not incorporated into SGs, and most of the SGs were not associated with PBs. These observations suggest that (1) SG–SG interactions are more common than SG–PB interactions and (2) in contrast to the report of Buchan et al. (2008), SGs can be assembled regardless of the presence of PBs in plants.

Most recently, single-molecule imaging of mRNA has provided direct evidence of mRNA localization and regulation under stress. Taking advantage of this approach, Wilbertz et al. (2019) designed three reporter mRNAs with different localization criteria under stress and tagged with MS2 stem-loops into the 3′ UTR to visualize. They observed that very few mRNAs directly move from SGs into PBs in human cells subjected to stress. Even in the reverse direction (from PBs to SGs), no events were detected during the experiment. Almost all the mRNAs were directly recruited to SGs or PBs, arguing against the proposed function of SGs as the sorting centers of mRNAs under stress. However, the authors did not rule out that other mRNAs, in addition to those tested, with particular *cis*-acting elements could be transferred between SGs and PBs. Indeed, Moon et al. (2019), employed the same MS2 stem loop-based technique but use *KDM5B* as a reporter gene, observing rapid bidirectional mRNA exchanges between SGs and PBs. This exchange suggests that there is no predefined pattern for mRNA movement between SGs and PBs.

Marondedze et al. (2020) investigated the changes in spliceosomal RNA-binding proteins under drought stress in *A. thaliana*. After co-expression analyses of spliceosome proteins with significant changes in abundance under drought stress, the authors identified 12 proteins that have been previously found as SG components. Remarkably, they reported that the protein binding to TOMV RNA 1L (BTR1L) is co-expressed with PAB4, PAB8, Rbp47a, and G3BP1. In addition, proteins PAB4 and PAB8 are shared between the spliceosome and SGs. The authors suggest that even when they are distant biological processes, a putative cross talk between spliceosome function and translational arrest may exist, which would be consistent with the identification of SG components. However, it needs to be further explored.

## Future Directions of SG Biology

Pioneering studies have established the baseline of our current understanding of SG biology, broadening our knowledge of the underlying mechanism of SG assembly and disassembly, composition, and structural organization. Many gaps in plant SG biology and dynamics still need to be addressed. Emerging and powerful optical microscopy techniques, such as super-resolution fluorescence microscopy and single-molecule imaging, will bring new insights into SG protein and RNA dynamics/structure in plant cells. Further, the study of the role of PTMs and small molecules as the modulators of SG assembly and disassembly will cope with the comprehension of SG dynamics.

A critical aspect in plant and non-plant models is the study of the specific components of the shell; the nature of PPI in the liquid phase represents a challenge for SG biology. Adapted PL in plants and cross-linked coupled with mass spectrometry techniques will clarify the differences in SG composition under different stress, cell types, and specific subcellular SGs. These findings will contribute to a better understanding of the biological role of plant SGs. Finally, most research on plant SGs has focused on how plants cope with different types of abiotic stress conditions. However, the composition and dynamics of plant SGs under biotic stress remain largely unexplored.

Nowadays, the world is facing linear increases in temperatures that have impacted tropical regions, such as West Africa; evidence reported the yield losses of ∼20% in 2019. Our ability to moderate and adapt plant stress responses will help ensure food security in the upcoming years. This is an appealing time for SG plant biology – there is a long road to drive, but every step along will generate biotechnological knowledge that will contribute to feeding the rapidly growing population.

## Author Contributions

IM-L, NF, IH-S, and MC contributed equally to the writing and review of this manuscript. All authors approved the final version of the manuscript.

## Conflict of Interest

The authors declare that the research was conducted in the absence of any commercial or financial relationships that could be construed as a potential conflict of interest.

## Publisher’s Note

All claims expressed in this article are solely those of the authors and do not necessarily represent those of their affiliated organizations, or those of the publisher, the editors and the reviewers. Any product that may be evaluated in this article, or claim that may be made by its manufacturer, is not guaranteed or endorsed by the publisher.
